# Bioelectrochemical Detection of *Mycobacterium tuberculosis* ESAT-6 in an Antibody-Based Biomicrosystem

**DOI:** 10.3390/s17102178

**Published:** 2017-09-22

**Authors:** Danna Sepulveda, Miguel A. Aroca, Andres Varela, Patricia Del Portillo, Johann F. Osma

**Affiliations:** 1 CMUA, Department of Electrical and Electronics Engineering, Universidad de los Andes, 111711 Bogota, Colombia; d.sepulveda10@uniandes.edu.co (D.S.); ma.aroca@uniandes.edu.co (M.A.A.); 2 CorpoGen, Carrera 5 No. 66A-34, 110231 Bogota, Colombia; avarela@corpogen.org (A.V.); pdelportillo@corpogen.org (P.D.P.)

**Keywords:** *Mycobacterium tuberculosis*, ESAT-6, biosensor, impedance, biomicrosystem

## Abstract

Bioelectrochemical sensing of *Mycobacterium tuberculosis* through electro-immunosensors is a promising technique to detect relevant analytes. In general, immunosensors require the formation of organic assemblies by the adsorption of molecular constituents. Moreover, they depend on the correct immobilization of the bio-recognition element in the biosensor. These procedures cannot be easily monitored without the use of invasive methods. In this work, an impedance analysis technique was used, as a non-invasive method, to measure and differentiate the manufacturing stages of the sensors. Biomicrosystems were fabricated through physical vapor deposition (PVD) of 80 nm Au nanolayers on 35 µm copper surfaces. Later, the surface was modified through thiolation methods generating a self-assembled-monolayer (SAM) with 20 mM 4-aminothiophenol (4-ATP) on which a polyclonal antibody (pAb) was covalently attached. Using impedance analysis, every step of the electro-immunosensor fabrication protocol was characterized using 40 independent replicas. Results showed that, compared to the negative controls, distilled water, and 0.5 µg/mL HSA, a maximum variation of 171% between each replica was achieved when compared to samples containing 0.5 µg/mL of ESAT-6 *M. tuberculosis* immunodominant protein. Therefore, this development validates a non-invasive method to electrically monitor the assembly process of electro-immunosensors and a tool for its further measure for detection of relevant antigens.

## 1. Introduction

Tuberculosis (TB) is a curable and preventable disease caused by the pathogenic bacterium *Mycobacterium tuberculosis*. Early diagnosis and treatment are essential measures for effectively controlling the epidemic disease [1]. However, TB detection remains a major obstacle due to several drawbacks of the available diagnostic methods, such as tuberculin skin test [2], bacteriological culture (e.g., mycobacteria growth indicator tube) [3], sputum smear microscopy, radiographic methods [4], and biochemical and nucleic acid amplification methods [5], among them. To date, the number of diagnosis approaches for TB has increased as the disease continues to be a mayor public health problem worldwide, as a result of the severe threat of multidrug-resistant (MDR) tuberculosis, extensively drug resistant TB (XDR) [6] and alarming frequency of TB among immunocompromised co-infected patients (e.g., HIV/AIDS) [7]. Nevertheless, most conventional detection technologies present difficulties in recognizing the presence of *M. tuberculosis*, since they are time consuming, do not provide clinically reliable results and significantly lack of sensitivity [8,9]. Furthermore, several techniques require various reagents and fluorescent or chemiluminescent labeling for sensing, they are usually low-yield and may include further purification steps [10,11,12]. Although TB mortality rate has been reduced by 47% since 1990, it is estimated that by 2014 there were 9.6 million new cases of TB [13]. Thus, this pathology has become the leading cause of death in people within the most economically productive age-groups and the second deadliest infectious disease in the world, generating misdiagnosis and poor treatment, especially in low and middle-income countries [14,15]. Since diagnosis represents a vital link in the TB control chain, new cost-effective detection platforms are required to achieve quality results in the shortest time span [16].

Biosensors have been introduced as promising technique for diagnostic due to their high sensitivity, short analysis time, cost-effectiveness and potential of miniaturization. Typically, biosensors are analytical devices that incorporate biological molecules (e.g., antibodies, enzymes, nucleic acids) as biorecognition probes in order to detect or interact with a target or analyte. Biomolecules can be immobilized in the sensor surface (e.g., glass or metal) via formation of self-assembled monolayers (SAMs) with thiols, disulfides, silanes, or acids [17]. For example, Kim et al. [2] developed immunocomplexes for detection of *M. tuberculosis* using silica-coated quantum dots and gold nanorods with antibodies as recognition elements. Wang et al. [18] fabricated an immunosensor on a screen printed carbon electrode for detection of the lipoarabinomannan antibody with the polysaccharide antigen as the detection probe. Torres-Chavolla and Alocilja [19] accomplished a biosensor with DNA probe for detection of TB IS6110 gene by isothermal amplification. The immobilization of biological molecules should ensure minimal steric hindrances interfering with the detected signal, which is provided by the interaction with the specific analyte [20]. Among biosensors, immunosensors are devices based on the antigen-antibody binding interaction to recognize the target for a later transduction into a readable signal.

Biosensing through the binding between the fragment antigen-binding (Fab) of the antibody with the epitope interface of the antigen is an affinity-based recognition, therefore, making possible the use of electrical circuitry for the transduction [21]. These type of biosensors, commonly-named electro-immunosensors, can detect with high selectivity based on the antigen-antibody affinity and, under the appropriate conditions, can be used without the need of lab facilities or highly-trained personnel [22]. Electrical stimulation and transduction of these type of biosensors can be carried out based on continuous signals (DC) (e.g., chronoamperometry, resistance), ramp signals (e.g., cyclic voltammetry), and sinusoidal signals (e.g., impedance analysis) between others. Among them, impedance analysis is based on the principle of measuring the changes in electrical properties of a conductive material due to the adsorption of an analyte on the surface functionalized with antibodies [23]. Contrary to other assays impedance biosensors can perform label-free detection, avoiding chemical amplification schemes, which include extra time, expense, and sample handling [24]. Recently, various impedimetric analysis platforms have been developed with high reproducibility. For instance, Jin et al. [25] developed a microfluidic impedimetric analysis system for the detection of the Cry1Ab protein. Ogata et al. [26] achieved label-free sensor for detection of human serum albumin by impedance with engineered virus particles. Sharma et al. [27] developed an electrochemical impedance sensor to quantify the binding of the human IL-8 with the immobilized probe. Wang et al. [28] reported a cardiomyocyte-based impedance biosensor for environment toxin detection. Matsishin et al. [29] fabricated a DNA-based impedimetric biosensor for detection of *rpoB* genes mutation of *M. tuberculosis*.

Electro-immunosensors integrated to fluidic microsystems allow the use of a minimum volume of sample and, therefore, of reagents, targeting feasible biomicrosystem diagnosis devices. With the inclusion of custom electronics, these biomicrosystems can be used as a non-destructive technique for early detection of TB without the need of specialized personnel, and their manufacturing process is already part of standard technology manufacture rather than highly-dependent lab procedures that are time-consuming and increment the diagnosis cost. Hence, in the present work, we designed, manufactured, and tested biomicrosystems based on industrial manufacturing processes as printed circuit board (PCB) platforms with 40 independent electro-immunosensors for the detection of the highly-expressed 6 kDa early secretory antigen target-6 (ESAT-6), an immunodominant secreted protein involved in the virulence of *M. tuberculosis* [30]. Human serum albumin (HSA) was employed to ensure that other molecules distinct to ESAT-6 does not bind to the sensor surface. Each electro-immunosensor is comprised of a gold nanolayer, and polyclonal antibodies (pAb) attached to the surface by a SAM created with thiols. Using impedance analysis at different frequency ranges, it was possible to detect probe-target interaction in different samples and, in addition, verifying all manufacturing stages of the biomicrosystems without the need of intrusive or destructive methods.

## 2. Materials and Methods

### 2.1. Reagents and Equipment

FR-4 (KB-6150) glass fabric slides of 142 mm × 48.7 mm, 0.7 mm thickness were obtained from Kingboard Laminates Ltd. (Hong Kong, China) and polymethylmethacrylate (PMMA) slides of 121 mm × 20.8 mm, 4 mm of thickness from Acrilcom (Uberlandia, Brasil). Gold at 99.9% was purchased from Kurt J. Lesker (Jefferson Hills, PA, USA), 4-aminothiophenol (4-ATP) at 97% and ethanol were obtained from Sigma Aldrich (Saint Louis, MO, USA) and phosphate buffered saline (PBS) was provided by Corpogen (Bogota, Colombia). The *M. tuberculosis* 6 kDa protein ESAT6 and the polyclonal antibody (pAb45073) were bought from Abcam (Cambridge, UK) and human serum albumin (HSA) was obtained from Biotest (Dreieich, Germany). A double-sided tape, TESA^®^ 4965 (TESA, Hamburg, Germany), and the electrical connectors were purchased at a local market. 

In order to calculate the pAb45073 detection limit we used different dilutions of ESAT-6 full length recombinant protein Abcam (ab124574) from 500 µg to 5 µg per ml in a dot blot assay. The assay included mouse monoclonal antibodies to ESAT6 Abcam (ab26246), goat anti-mouse IgG H and L secondary antibody conjugated to horse radish peroxidase (HRP) and HRP substrate TMB (3,3’,5,5’-tetramethylbenzidine) to detect the antigen antibody interaction.

PCB was fabricated with dry film photopolymer LAMINAR^®^ E9200 that was obtained from Eternal Materials Co Ltd (Kaohsiung, Taiwan), Dynamask KM, developer for negative presensitized boards and stripper for positive and negative photoresists were acquired from Bungard Elektronik (Windeck, Germany). Negative films were printed with the Filmstar Photoplotter (Bungard Elektronik, Windeck, Germany). The adhesion of the photosensitive film was performed with a RLM 419P dry film laminator and the UV exposure was achieved with the double-sided Hellas exposure unit (Bungard Elektronik, Windeck, Germany). The wet processes of circuit manufacture were carried out at the Splash Center (Bungard Elektronik, Windeck, Germany).

The physical vapor deposition of gold was accomplished with a Edwards Auto 306 physical thermal evaporator (Moorfield, Knutsford, UK) and the biomicrosystem measurements were carried out with an Agilent 4294A impedance analyzer (Agilent Technologies, Santa Clara, CA, USA) and a PeakTech 3725 multifunction tester (PeakTech, Ahrensburg, Germany). The immobilized surface observations were performed with a Phenom G2 pro desktop scanning electron microscope (SEM) (Phenom-World, Eindhoven, The Netherlands).

### 2.2. PCB Manufacturing

For the biomicrosystem base a double-layer FR-4 PCB with 2 oz thickness of copper was designed and manufactured. The electrode pattern was made with CadSoft Eagle Professional 7.4.0. The PCB bottom surface was laminated with antisolder and the top layer was left uncovered for further material deposition. Subsequently, a uniform gold layer was physically evaporated onto the exposed electrodes of this surface. Finally, the PCB electrical conductivity was verified through PeakTech 3725 multifunction digital tester (PeakTech, Ahrensburg, Germany).

### 2.3. Lift off and Gold Deposition

The substrate top surface was laminated with a dry photosensitive film (LAMINAR^®^ E9200) in the RLM 419P (Bungard Elektronik, Windeck, Germany) and then to imprint the electrode layout substrate was exposed to UV light with the printed electrode layout. The laminated substrate was then subjected to a developing process to remove the exposed photosensitive layer with the developer (Bungard Elektronik, Windeck, Germany). Afterwards, a uniform gold nanolayer was physically evaporated on the laminated substrate through PVD with a thermal evaporator. A 3 A current was used over a tungsten slide for the 100 mg gold evaporation. A vacuum pressure of 4 × 10^−5^ mbar and an evaporation rate of 0.12 nm/s were established, thus obtaining 80 nm Au nanolayers on 35 um copper surfaces.

Finally, the residual photoresist was removed with a stripper (Bungard Elektronik, Windeck, Germany), so that the electrode pattern was settled onto the card. Finally, the biomicrosystem electrical conductivity of individual electrodes on the board was verified using a PeakTech 3725 multifunction digital tester (PeakTech, Ahrensburg, Germany).

### 2.4. Biomicrosystem Development

**Figure 1:**
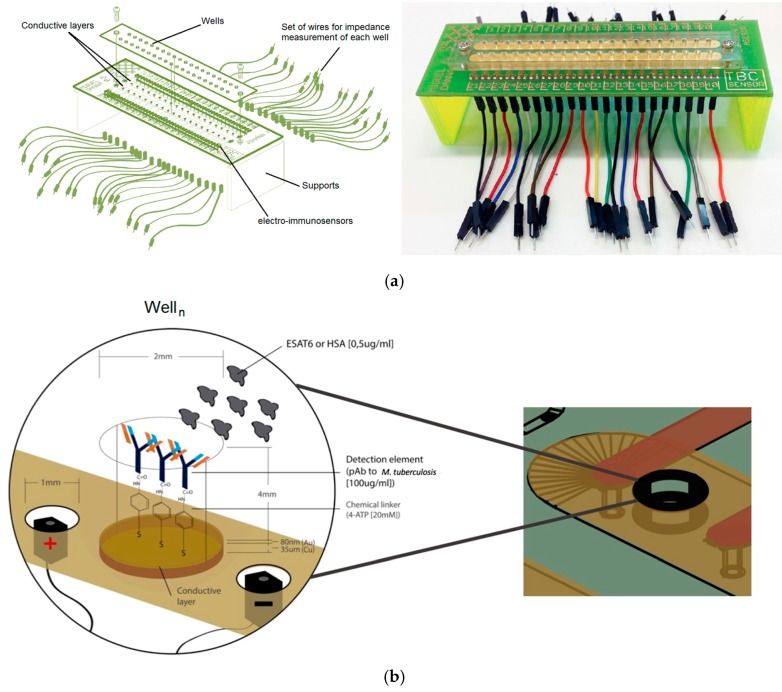

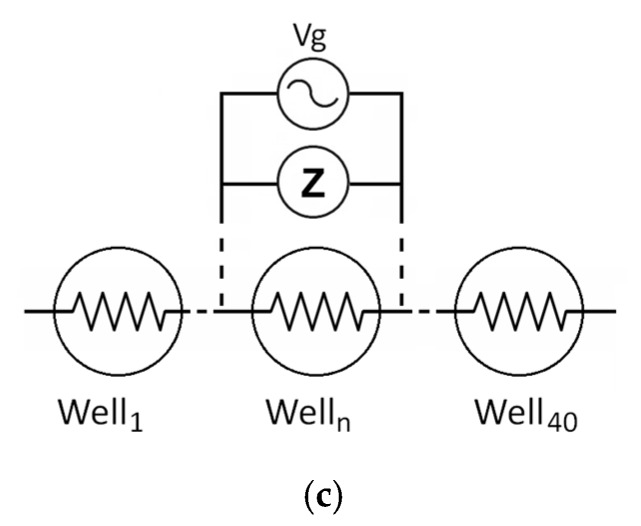
(**a**) Left: schematic representation of the biomicrosystem. Right: Photo of one of the biomicrosystems fabricated; (**b**) schematic illustration of one electro-immunosensor well showing the reagent assembly and capture probe layer; and (**c**) an illustration of the measuring method for each electro-immunosensor of the biomicrosystem, where Vg is the generator and Z the impedance meter, both part of the impedance analyzer.

On the substrate covered with gold a 4 mm PMMA slide with 2 mm wells organized in two rows and 20 columns was stuck to the top through the use of TESA^®^ 4965 double-sided tape, miming a well plate with individual wells for each electrode. Inside each well reagents were deposited for the biosensor fabrication. Between each well, electrical connectors were placed for electrical measurements. A total of three biomicrosystems were fabricated in different batches and tested, each manufactured biomicrosystem contained 40 independent electro-immunosensors for a total of 120 electro-immunosensors, that had to be measured individually (Figure 1) to verify the reproducibility and repeatability of the manufacture process.

### 2.5. Reagents Immobilization

For the self-assembled monolayer (SAM) fabrication 4-ATP molecules were added as cross-linkers for the antibodies in each well. A previously-reported electro-immunosensor by our group [22] allowed us to conclude that the best concentrations for building up a self-assembled monolayer were 20 mM of 4-ATP and 100 µg/mL of antibody. Therefore, a solution of 10 µL of a 20 mM 4-ATP was added into the wells. Later, after 4 h at room temperature, the wells were washed with ethanol and deionized water, in order to remove the remaining thiols. Then 10 µL of 100 µg/mL pAb 45073 solution was added into each well and the system was incubated at 4 °C over night. Finally, the remaining biomolecules were washed with PBS and deionized water.

### 2.6. Immobilization Testing by Electrical Techniques

Electro-immunosensor characterization was made through impedance measurements that were performed in each well with an impedance analyzer (Agilent 4294A) at a frequency range from 40 to 280 Hz. Measurements at particular frequencies were performed by eight replicates for the three manufacturing stages of the sensors: gold nanolayer (Au), gold nanolayer with a SAM of 4-ATP (Au + ATP), gold nanolayer with a SAM of 4-ATP attaching pAb 45073 (Au + ATP + pA), and for two analyte configurations: in the presence of 0.5 µg/mL of ESAT6 (Au + ATP + pA-ESAT6) (positive control) and in the presence of HSA (Au + ATP + pA-HSA) (negative control), ten times lower than the detection limit obtained using dot blot assay. Incubation of ESAT-6 or 0.5 µg/mL of HSA in each well took place at room temperature for 1 h under static conditions prior to electrical measurements.

### 2.7. Analytic Hierarchy Process for the Biomicrosystem and Traditional Detection Techniques

The analytic hierarchy process (AHP) was used as a decision-making tool to study the performance of the electro-immunosensor in contrast with conventional TB detection systems, such as Xpert MTB/RIF (Cepheid, Sunnyvale, CA, USA), culture, and sputum smear test [31]. The decision-making criteria considered includes cost, effectiveness, time, and the test analysis process. Cost involves the commercial expenses of performing each analysis, which were assumed based on the mean international costs reported by international laboratories, while the cost of the biosensor was calculated based on its manufacturing procedure; effectiveness is the assay capacity to accomplish an intended result under the possibility of noise; and time refers to the speed of analysis, and the analysis of the tests is subdivided according to the following two subcriteria: liability of the analysis under contamination hazard and viability on the assumption of blood in the samples.

The pairwise comparison matrix was constructed on the basis of tuberculosis experts consensus and taking into account the 1 to 9 scale criteria proposed by Saaty [32]. Expert Choice 11.5^®^ (ExpertChoice, Arlington, VA, USA) software was used to rank the alternatives of the decision with respect to the criteria or subcriteria.

## 3. Results

### 3.1. Immobilization Testing by Electrical Techniques

**Figure 2:**
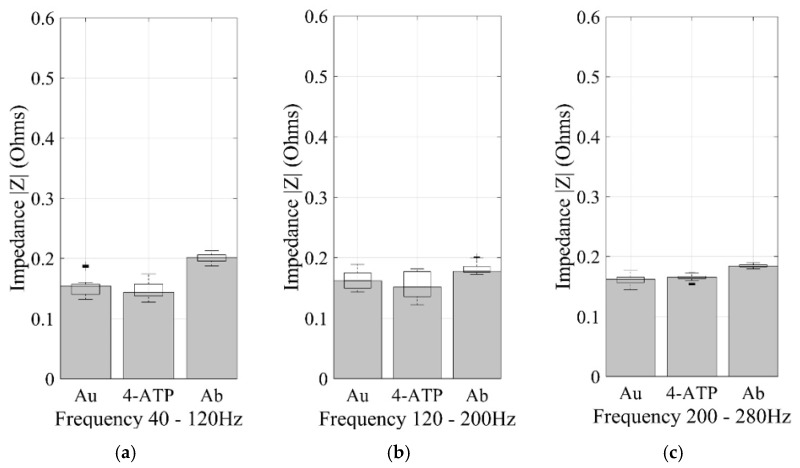
Analysis of impedance variation for manufacturing stages. (**a**) The impedance magnitude at 40–120 Hz; (**b**) the impedance magnitude at 120–200 Hz; and (**c**) the impedance magnitude at 200–280 Hz.

**Figure 3:**
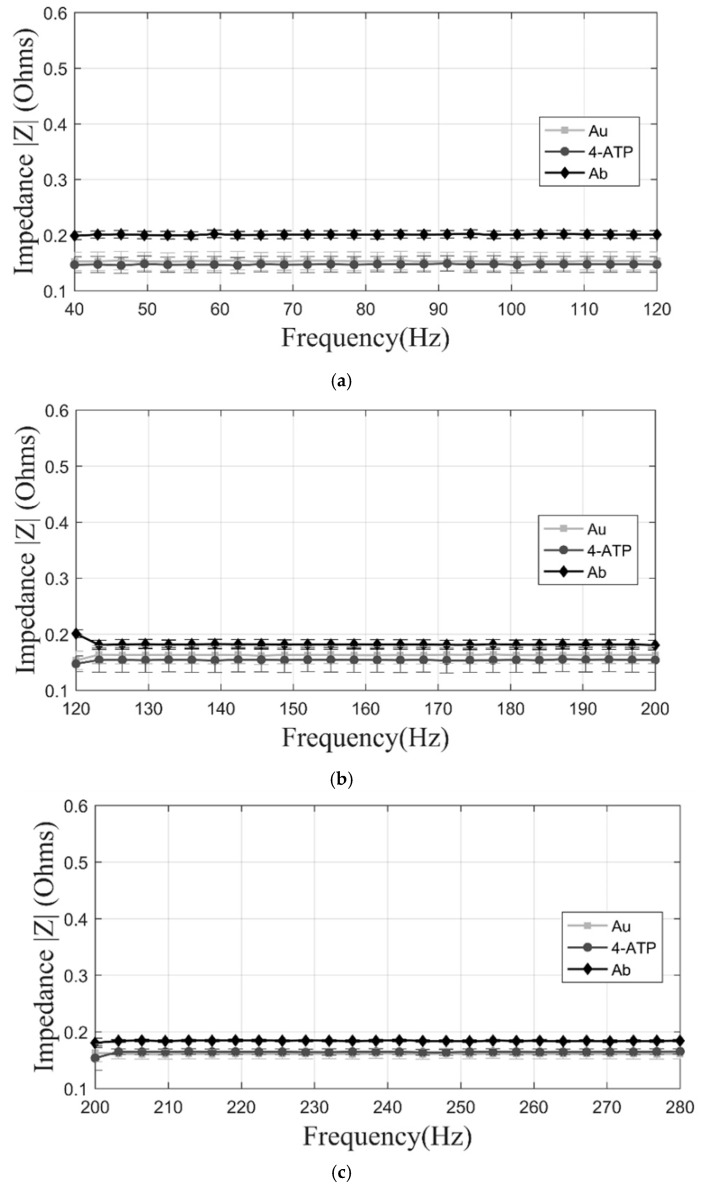
Linear plot of impedance values of the manufacturing stages. (**a**) The impedance magnitude at 40–120 Hz; (**b**) the impedance magnitude at 120–200 Hz; and (**c**) the impedance magnitude at 200–280 Hz.

**Figure 4:**
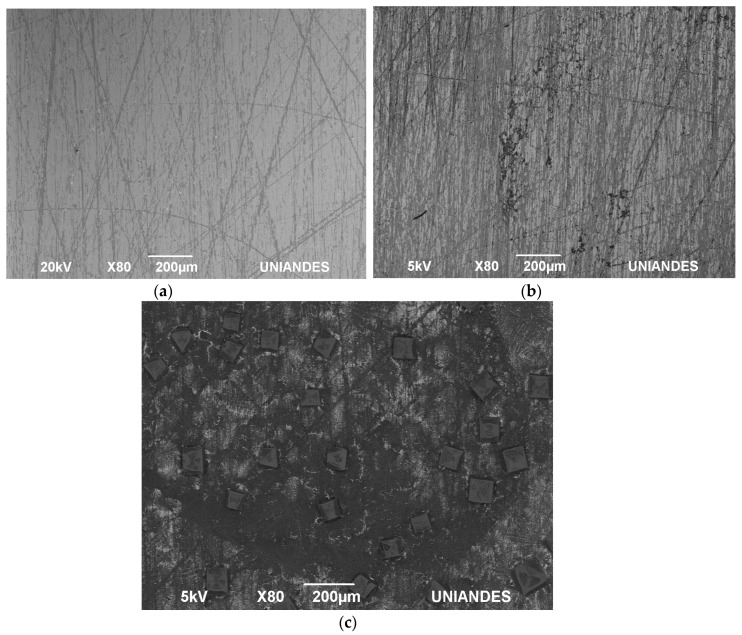
Observation of samples in the SEM: (**a**) Au; (**b**) Au + ATP; and (**c**) Au + ATP + pA.

The average impedance magnitude of the three manufacturing stages of the sensors was determined in response to an AC current as a function of frequency [33]. The characterization of the replicas of different stages of immobilization within the biosensor led to determining a range of measurement frequencies with low noise, allowing the replicability of the experiment. In order to determine the variation of the copper with gold evaporated prior to the immobilization (conductive layer), 40 individual wells of three biomicrosystems fabricated in different batches, for a total of 120 samples, were measured by impedance analysis at different frequency ranges. The mean value of the 120 wells in the range of 40 to 120 Hz was 0.1588 Ω with a standard deviation of 0.0142. In the range of 120 to 200 Hz, the mean value was 0.1591 Ω with a standard deviation of 0.0142. Finally, in the range of 200 to 280 Hz, the mean value was 0.1584 Ω with a standard deviation of 0.0102 revealing a small variance in the manufacture of all wells independently from the fabrication batch. When comparing the results between fabrication batches, the maximum impedance variation between the biomicrosystems was 0.43% in the frequency range of 40 to 120 Hz, compared to 0.41% and 0.06% of differences in the 120 to 200 Hz and 200 to 280 Hz frequency ranges, showing an appropriate reproducibility of the manufacturing process. As it can be seen in the plots of Figure 2 and Figure 3, the range where the largest significant differences were found within immobilization stages was between 40 and 120 Hz. In this range, there is a non-significant variation of impedance (−3.81%) between the first two manufacturing stages, Au and Au + ATP. Moreover, there is an impedance variation of 31.21% between Au and Au + ATP + pA. On the other hand, from 120 to 200 Hz there is an impedance variation of (−5.51%) between the first two manufacturing stages and an 11.44% impedance variation between Au and Au + ATP + pA. Finally, at frequencies of 200 to 280 Hz we measured a 2.16% impedance variation between the first two manufacturing stages and 14.37% between Au and Au + ATP + pA. The highest impedance value of the last immobilization step was 0.20 Ω. Surface observation in the SEM (Figure 4) showed a non-homogeneous structure and a change in the surface of the three stages (Au, Au + ATP, Au + ATP + pA).

### 3.2. ESAT6 Detection

**Figure 5:**
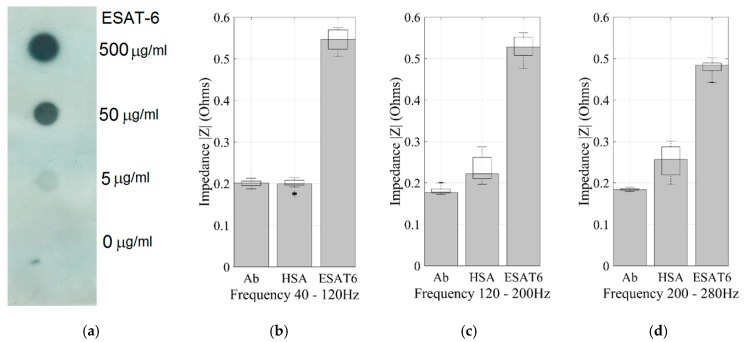
(**a**) Dot blot assay as a comparison to the electro-immunosensor detection limit. Analysis of impedance variation for 0.5 µg/mL of analyte configurations of the sensors; (**b**) the impedance magnitude at 40–120 Hz; (**c**) the impedance magnitude at 120–200 Hz; and (**d**) the impedance magnitude at 200–280 Hz.

**Figure 6:**
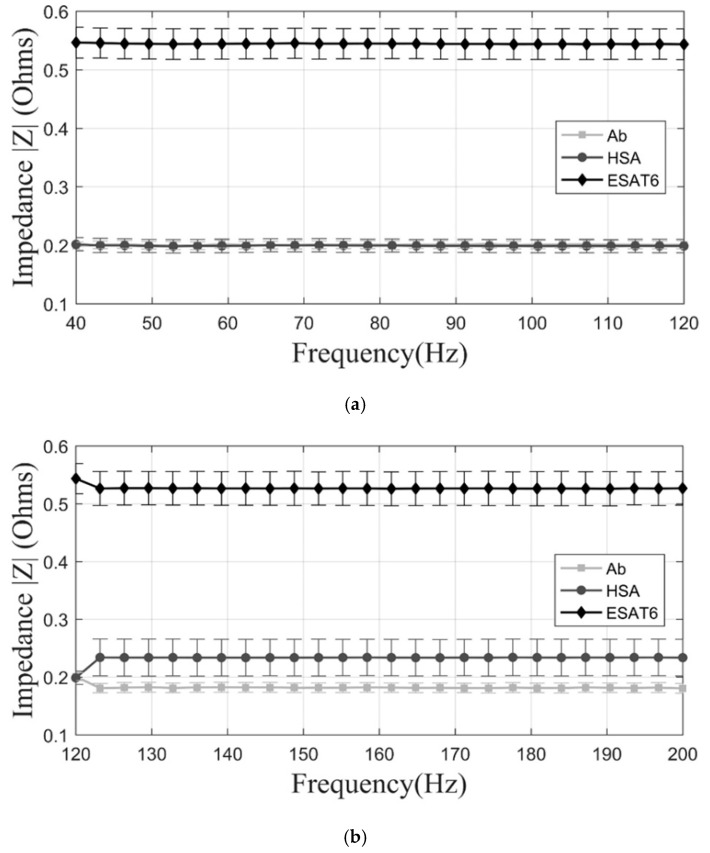

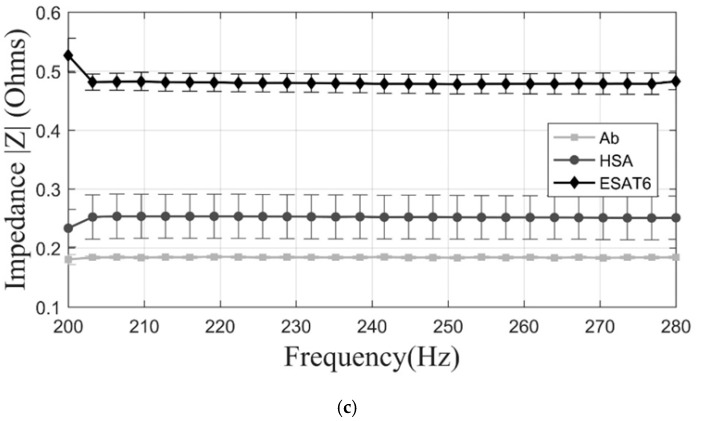
Linear plot of impedance values of the analyte configurations of the sensors. (**a**) The impedance magnitude at 40–120 Hz; (**b**) the impedance magnitude at 120–200 Hz; and (**c**) the impedance magnitude at 200–280 Hz.

The performance of the biosensor for the detection of a positive and negative control, 0.5 µg/mL ESAT-6 and 0.5 µg/mL HAS, respectively, was verified through impedance measurements and compared to the dot blot assay. Figure 5 and Figure 6 show that the frequency range from 40 Hz to 120 Hz has the most significant variation in impedance among all the investigated ranges. The detection of the *M. tuberculosis* protein has a difference of 171% in impedance increase compared to the impedance measured for the negative control (HSA) in this frequency range (Figure 6A). The negative control indeed produced a null impedance change compared to the biosensor impedance with only distilled water, ruling out the possibility of misleading interpretations of the results. Figure 6B,C show that there is a significant change in other frequency ranges (120–200 Hz and 200–280 Hz), however, the greatest impedance variation can be found in the range of 40–120 Hz. Regarding Figure 5, it is important to notice that there are no overlapping values between the negative and the positive control. We observed that all the results of the negative controls were under 0.20 Ω and that impedance values for the positive control were all above 0.54 Ω. Thus, we establish a decision range where results in the grey zone where the protein presence is unreliable and additional evaluation of the sample needs to be done. Finally, at 40 Hz we verified that 100% of the performed proof with ESAT-6 protein were successful, demonstrating the efficient performance of the electro-immunosensor at low frequencies.

### 3.3. Analytic Hierarchy Process for the Biomicrosystem and Traditional Detection Techniques

Judgement results of the confrontation between the electro-immunosensor and traditional TB diagnostic alternatives (Xpert MTB/RIF, culture, and smear test) were evaluated using Expert Choice 11.5^®^ software. Table 1. Documents the information for the construction of the pairwise comparison matrix of cost, effectiveness, time, and the test analysis. The comparative judgement was made in Expert Choice^®^ 11.5 in order to weigh the priorities of the alternatives in respect to the criteria. The total weighted value for each criterion was 100% and was distributed among the four diagnostic alternatives. Local weights obtained for the electro-immunosensor, Xpert MTB/RIF, culture and smear test were: for commercial costs, 31%, 4.3%, 11.3%, and 53.4%; for analysis of the tests, 35.6%, 15.8%, 28.1%, and 20.5%; for the effectiveness, 10.6%, 14.4%, 54.3%, and 20.8%; and for the time of analysis, 50.2%, 29%, 4.1%, and 16.7%, respectively. In short, the global weights obtained for each assay were 32.5%, 16.7%, 22.5%, and 28.3%, respectively.

According to the results obtained by evaluating the AHP alternatives in Expert Choice^®^ 11.5, the electro-immunosensor was found to be the dominant alternative, globally, in comparison with the traditional alternatives for TB detection. Moreover, it was determined to be locally dominant in the speed of analysis criteria, since the device requires less time to obtain the results among all the evaluated assays. In addition, a single device allows to characterize 40 independent replicas, detecting several *M. tuberculosis* epitopes, simply by changing the bio-recognition probe in each biosensor. Furthermore, the analysis liability is not altered by the presence of bleeding due to antigen affinity-based recognition by the antibody. In the chart, the electro-immunosensor did not exhibit local dominance with respect to the criteria commercial costs, as a result of the bioreagent cost. Even though the use of the antibody has a significant cost, the manufacturing cost of each biosensor is very low compared to other automated and non-automated techniques. The device did not have local dominance in the assay effectiveness criteria, since extra experimental studies with clinical samples are required to establish a standardized procedure to diagnose TB, minimizing noise.

## 4. Discussion

The designed biomicrosystem shows promising results as a label-free detection device for *M. tuberculosis* epitopes, this enables patient treatment in a quicker and more affordable platform to control TB outbreaks. In each fabricated device it is possible to perform 40 independent tests using small amounts of reagents, each probe is covalently attached to a thiol to enhance the biomolecular stability. Moreover, depending on the selected probes it is possible to detect different analytes in the electro-immunosensor. The device does not require special reagents, simplifying the diagnosis of the pathology, which is normally laborious and also requires trained personnel and specialized infrastructure.

In TB detection, the speed of analysis is a crucial factor because for delay decisions patients remain untreated. The electro-immunosensor takes approximately an hour to have a confirmatory result in each biosensor. In short, the device could allow early detection of the disease and the rapid onset of treatment avoiding problems of desertion, drug resistance, morbidity or mortality of the patient. In contrast to other TB diagnostic tools, each electro-immunosensor is less expensive than standard techniques, costing approximately $7 USD. Moreover, it takes less time detecting biomolecules and it does not require specialized personnel and infrastructure compared to traditional technologies, such as culture, smear test, and nucleic acid amplification tests. Although experiments with clinical samples are yet to be developed, ESAT-6 is an abundantly-secreted antigen. Therefore, one of the best antigens to detect, giving potentially good results when detected with the electro-immunosensor. In addition, all stages used in the fabrication of the electro-immunosensor can be easily scaled up since all processes are based on industrial manufacturing technologies.

## 5. Conclusions

The biosensing miniaturized platform can be used for ESAT-6 detection in about 1 h of analysis time. Standard PCB technology used to manufacture each biosensor optimizes the time, cost, and replicability of the fabrication, the parameters of major relevance in TB diagnosis. The manufacturing technology of the biosensor makes it possible to build a standard device with a simplified use for the operator. On the other hand, it was possible to identify the assembling process within the biosensor interface through impedance measurements, made for the three manufacturing stages with the electro-immunosensor, from 40 to 280 Hz. Measurements exhibited less impedance deviation at 40 Hz; at that frequency it is possible to monitor the surface activity, enhancing both the sensitivity and specificity of the biosensor. The study and analysis of the biosensor performance with positive and negative controls allowed the identification of a decision range for the interpretation of the results. ESAT-6 presence can be noticed above 0.54 Ω and non-presence below 0.20 Ω, between 0.20 Ω and 0.54 Ω, there is a gray zone where measurements must be repeated. Moreover, with a small quantity of reagents impedance variation between positive and negative control is 171%. Finally, further studies are in progress to enhance the biomicrosystem as a multiplexed point-of-care device with a portable impedance analyzer, which can be used for the automated detection of several antigens.

## Figures and Tables

**Table 1:** Criteria for the establishment of the pairwise comparison matrix for the diagnostic techniques for *M. tuberculosis*.

Test	Cost	Time of Analysis	Probability of Contamination	Difficulty of Analysis for Bleeding Presence	Test Effectiveness
**TBC biosensor**	$7 USD (no labor and overhead cost)	1 h	Low: Although it is a manual process, there are mechanical barriers that avoid reagent filtration between wells	It is based on antigen-antibody binding, so it is not supposed to affect the results	The results confirm that reliable data is obtained with the biosensor
**Xpert MTB/RIF**	$98.10 USD	2 h	None: Completely automated	With pretreatment the presence of bleeding that can alter the analysis is insignificant	High sensitivity, specificity and reproducibility. Avoid false positive/negative results
**Culture (MGIT)**	$35.56 USD	One month	Medium: Microbial growth can be affected by accompanying microbiota	Bleeding presence does not alter the analysis	Total reliability (70–90%)
**Smear microscopy test**	$4.07 USD	More than one day	None: it is a fast and direct method	With pretreatment the presence of bleeding that can alter the analysis is insignificant	It depends on the sample and the investigator. It has a variable sensibility range 22–80%
